# Duplication of 4-bp in *SACS* leads to autosomal recessive spastic ataxia of Charlevoix–Saguenay type in two Pakistani patients

**DOI:** 10.1038/s41439-026-00349-z

**Published:** 2026-04-28

**Authors:** Saba Bibi, Asad Munir, Fawad Ali, Helen Nabiryo Frederiksen, Sabawoon Shah, Abdur Rashid, Sergey Oreshkov, Shahab Uddin, Hamid Ur Rahman, Shumaila Noreen, Mukhtar Ullah, Muhammad Ansar, Atta Ur Rehman

**Affiliations:** 1 Department of Zoology, Faculty of Biological and Health Sciences, Hazara University, Mansehra, Pakistan; 2 Department of Neurology, Saidu Group of Teaching Hospital, Saidu Sharif, Swat Pakistan; 3 Department of Ophthalmology, University of Lausanne, Jules Gonin Eye Hospital, Fondation Asile Des Aveugles, Lausanne, Switzerland; 4 Department of Medicine, Luqman International Hospital, Saidu Sharif, Swat Pakistan; 5 Advanced Molecular Genetics and Genomics Disease Research and Treatment Centre, Dow University of Health Sciences, Sindh, Pakistan

**Keywords:** Medical genetics, Neurodegeneration

## Abstract

In this study, we present two patients from a Pakistani family affected by autosomal recessive spastic ataxia of Charlevoix–Saguenay, a rare neurodegenerative disorder. Exome sequencing identified a homozygous 4-bp duplication (NM_014363.6:c.12129_12132dup, p.Leu4045ArgfsTer8) in *SACS* correlating with disease in the affected family members.

ARSACS, initially discovered in the population from the Charlevoix–Saguenay Lac-Saint-Jean (CSLSJ) region in Quebec, is an early-onset neurodegenerative disorder affecting ~2.7 individuals per 100,000 live births globally^1,2^. Patients with ARSACS typically display cerebellar ataxia, peripheral neuropathy, and pyramidal tract signs with the condition leading to dependency on wheelchair in most patients^1,3^. Upon electrophysiological examination, patients often show moderately reduced motor nerve conduction velocities, whereas brain MRI studies show superior vermis atrophy, linear hypointensities in the pons, and atrophy of the cerebellar hemispheres and spinal cord^4,5^. The *SACS* gene, located on 13q12.12 and spanning 11 coding exons, encodes Sacsin, a protein believed to contain the UbL domain at the N terminus, a DnaJ domain, and a HEPN domain at the C terminus^1,6,7^. Expression of *SACS* is remarkable in the central nervous system, although its expression in other tissues such as skin, skeletal muscles, and pancreas has also been noted. OMIM associates *SACS* with spastic ataxia, Charlevoix–Saguenay type (MIM no. 270550), inherited as an autosomal recessive entity. Sacsin is an ataxia protein and a regulator of the Hsp70 chaperone machinery that has been implicated in the processing of other ataxia-linked proteins^7^.

In this study, a consanguineously married nuclear family with four children, including two affected (MA1080 and MA1081), was presented for molecular diagnosis as shown in Fig. 1A. During our last examination performed in 2022, affected brothers were 27 and 22 years old. Progressive generalized body weakness with wasting, progressive walking difficulty with increased falls, and scanning speech were noted in both brothers as early as age 4. On clinical examinations, the patients appeared to have hammer toe, swan neck deformity in hand, pes cavus, brisk knee reflexes, and absent ankle reflex, with few fasciculations, positive upper motor neuron (UMN) signs and positive Babinski sign, gait ataxia, distal amyotrophy, and proximal and distal muscle weakness. Moreover, ocular examinations showed bilateral horizontal nystagmus, impaired smooth pursuit, and hypermyelinated retinal fibers. On the basis of these symptoms, patients were initially identified to use ankle foot orthosis and referred to physiotherapy. As shown in Fig. 2, electromyography (EMG) and nerve conduction studies (NCSs) in the proband were suggestive of chronic inflammatory demyelinating polyradiculopathy with laboratory findings showing normal serum calcium and phosphate levels. MRI brain and X-ray radiographs were unremarkable. On the follow-up visit, there was worsening of symptoms and no substantial clinical improvement. Diagnosis was reconsidered as patients did not fit into the chronic inflammatory demyelinating polyradiculopathy as they were having brisk reflexes and UMN signs. A differential diagnosis of spinocerebellar ataxia (SCA) was considered. On follow-up visit, patients had complaints of clinical SCA with features of polyneuropathy and mild bulbar symptoms for which they were referred to physiotherapists. On the next follow-up visit, patients complained of progressive generalized muscle weakness mainly in proximal and distal muscles and positive UMN signs with scanning speech and was advised for cerebrospinal fluid, NCS and EMG. Upon evaluation of the NCS/EMG findings (Fig. 2a–p), left median and right median motor nerves showed prolonged distal onset latency (L 8.9 ms and R 12.6 ms), reduced amplitude (L 0.7 mV and R 1.0 mV), and decreased conduction velocity (elbow-wrist, L 36 m/s and R 28 m/s). The left peroneal motor and the right peroneal motor nerves showed prolonged distal onset latency (L 13.8 ms and R 13.8 ms) and mildly reduced amplitude (L 0.0 mV and R0.0 mV). The left tibial motor and the right tibial motor nerves showed prolonged distal onset latency (L 13.8 ms and R 13.8 ms) and reduced amplitude (L 0.0 mV and R 0.1 mV). The left ulnar motor nerve showed prolonged distal onset latency (7.9 ms) and decreased conduction velocity (B elbow-wrist, 21 m/s). The right ulnar motor nerve showed prolonged distal onset latency (9.5 ms), reduced amplitude (1.1 mV), decreased conduction velocity (B elbow-wrist, 35 m/s), and decreased conduction velocity (A elbow–B elbow, 37 m/s). The right median 2nd digit sensory and the right radial sensory nerves showed prolonged distal peak latency (R 4.0 ms and R 3.4 ms) and reduced amplitude (R 6.5 μV and R3.9 μV). The left median 3rd digit sensory and the right median 3rd digit sensory nerves showed prolonged distal peak latency of palm (L 2.3 ms and R 5.3 ms) and wrist (L 5.7 ms and R5.5 ms). The left radial sensory nerve showed prolonged distal peak latency (4.8 ms). The left ulnar sensory and right ulnar sensory nerves showed prolonged distal peak latency (L 6.1 ms and R5.3 ms), reduced amplitude (L 9.2 μV and R4.2 μV), and decreased conduction velocity (wrist-5th digit, L 28 m/s and R 32 m/s). All remaining nerves were within normal limits. After no significant clinical improvement, a re-workup was considered along with genome sequencing.

**Fig. 1:**
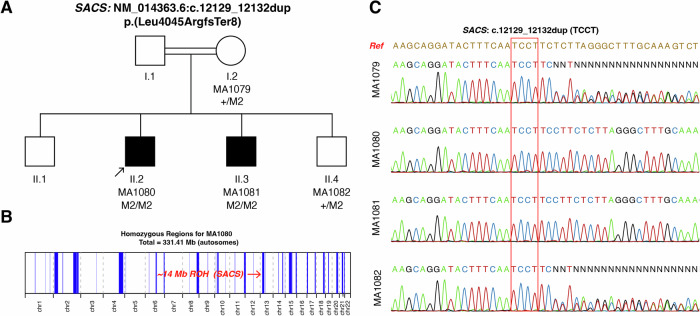
Familial genotype-phenotype correlation analysis and homozygosity mapping. **A**, A two-generation nuclear family with a double horizontal line between parents indicating consanguinity. Proband (II.2) in the pedigree is pointed by an arrow. Dark-filled symbols represent patients, whereas blank symbols show healthy individuals in the family. + symbolizes wild-type allele, whereas “M“ represents identified mutation [SACS:c.12129_12132dup]. **B**, Blue vertical peaks along horizontal axis mean runs of homozygosity (ROH) at individual chromosomes (chromosome nos 1–22). *SACS* gene has been found inside ~14-Mb long ROH on chromosome 13 as indicated by an arrow in red color. **C**, Sanger chromatograms of the available family members. Double peaked chromatograms of the proband’s mother (MA1079) and his one available healthy brother (MA1082) confirm their status to be both heterozygous carriers, whereas single peaked chromatograms for both patients (MA1080 and MA1081) confirm that they were both homozygous for the identified mutation.

**Fig. 2:**
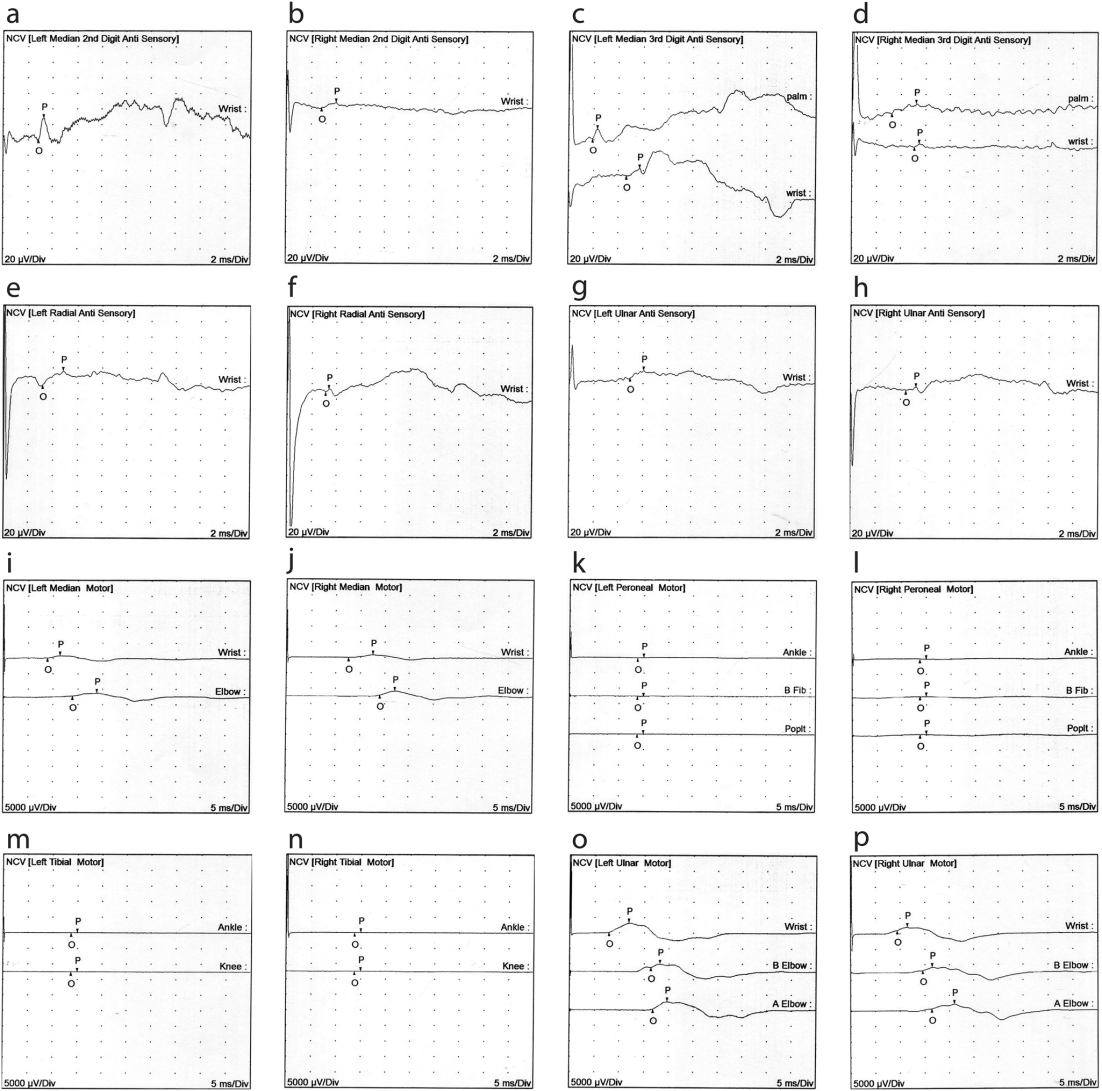
Abnormal electromyography and nerve conduction studies of the proband suggesting chronic inflammatory demyelinating polyradiculopathy. **a**,**b**, Waveform of left median 2nd digit antisensory and right median 2nd digit antisensory nerves showed prolonged distal peak latency (R 4.0 ms and R 3.4 ms) and reduced amplitude (R 6.5 μV and R 3.9 μV). **c**,**d**, Left median 3rd digit antisensory and right median 3rd digit antisensory nerves showed prolonged distal peak latency (palm, L 2.3 ms and R 5.3 ms) and prolonged distal peak latency (wrist, L 5.7 ms and R 5.5 ms). **e**, Left radial antisensory within normal limit. **f**, Right radial antisensory within normal limit. **g**, Left ulnar antisensory within normal limit. **h**, Right ulnar antisensory within normal limit. **i**,**j**, Left median motor and right median motor nerves showed prolonged distal onset latency (L 8.9 ms and R12.6 ms), reduced amplitude (L 0.7 mV and R 1.0 mV), and decreased conduction velocity (elbow-wrist, L 36 m/s and R 28 m/s). **k**,**l**, Left peroneal motor and right peroneal motor nerves showed prolonged distal onset latency (L 13.8 ms and R 13.8 ms) and mildly reduced amplitude (L 0.0 mV and R 0.0 mV). **m**,**n**, Left tibial motor and right tibial motor nerves showed prolonged distal onset latency (L 13.8 ms and R 13.8 ms) and reduced amplitude (L 0.0 mV and R 0.1 mV). **o**, Left ulnar motor nerve showed prolonged distal onset latency (7.9 ms) and decreased conduction velocity (B elbow-wrist, 21 m/s). **p**, Right ulnar motor nerve showed prolonged distal onset latency (9.5 ms), reduced amplitude (1.1 mV), decreased conduction velocity (B elbow-wrist, 35 m/s), and decreased conduction velocity (A elbow–B elbow, 37 m/s). NCV, nerve conduction velocity.

Exome sequencing identified a homozygous 4-bp duplication in the final exon of the *SACS* gene (NM_014363.6:c.12129_12132dup). The variant was predicted to cause frameshift, thereby introducing a premature termination codon (p.Leu4045ArgfsTer8). As the variant is located in the final exon of the gene, it is less likely to trigger nonsense-mediated mRNA decay. However, the variant may lead to a C-terminally truncated Sacsin protein, lacking in last 528 amino acids. Sanger sequencing confirmed that the variant was homozygous in both patients (MA1080 and MA1081), whereas the variant was heterozygous in patients’ mother (MA1079) and one healthy brother (MA1082) (Fig. 1C). Duplication occurred inside a highly conserved region of the genome (PhyloP100 score = 7.841). The variant is novel and has never been previously reported in ClinVar or other clinical variant databases, including the Human Gene Mutation Database (accessed January 18, 2026). On the basis of the American College of Medical Genetics and Genomics guidelines, the variant was classified as pathogenic with the criteria PVS1 as strong, PM2 as moderate, PM3 as moderate, PP1 as supporting, and PP4 as supporting. Homozygosity mapping located *SACS* inside a 14-Mb homozygous interval on chromosome 13, whereas a cumulative 331.41-Mb of autosomal genome was found to be homozygous (Fig. 1B). Thus, our homozygosity mapping results reiterated parental consanguinity as shown in the pedigree (Fig. 1A). Extensive clinical overlap exists between symptoms of cerebellar ataxia and other spastic ataxias and spasticity, thus challenging a differential diagnosis among them^5^. For instance, our patients were diagnosed with hereditary spastic cerebellar ataxia (HSCA) after a series of clinical evaluations. Unfortunately, however, the family remained without molecular diagnosis for many years. As more than 50% marriages in Pakistan are consanguineous^8,9^, we predict that a sizeable proportion of Pakistani families with HSCA and other monogenic conditions may still await molecular diagnosis owing to lack of infrastructural support and limited resources.

As of today, *SACS-*related HSCAs have been identified in eight Pakistani families including six families from Khyber Pakhtunkhwa and two families from Punjab^5,10–13^. Across these families, eight distinct *SACS* variants were identified including one missense (p.Arg1645Pro), three frameshift (p.Phe743LeufsTer8, p.Asn1586TyrfsTer3, and p.Asn3040 LysfsTer4) and four nonsense variants (p.Arg88Ter, p.Arg401Ter, p.Arg728Ter, and p.Gln886Ter). In line with our findings, all previously identified *SACS* variants in Pakistani families were found in homozygous state. These findings underscore the role of consanguinity in the prevalence of rare monogenic conditions in the Pakistani population^14,15^. Of the published Pakistani HSCA cohort, index patients in two families were reported to be completely paralyzed by age 26 and 15 years^5,11^. Although our patients were not completely paralyzed at last clinical evaluation, a progressive HSCA was evident in both brothers showing typical clinical presentation in ARSACS cases. Contrary to previous reports^5,12,13,16^, proband’s brain MRIs were unremarkable except for the presence of mucus retention cyst in the left maxillary sinus and mucosal thickening in the right maxillary sinus. Conversely, NCS and EMG findings in our proband were abnormal, thus supporting literature^11,16^. In summary, we report two brothers of Pakistani descent affected by a rare neurodegenerative disorder owing to a 4-bp duplication in *SACS*. Further studies are required to fully elucidate the impact of the identified variant on protein structure/function.

## HGV Database

The relevant data from this Data Report are hosted at the Human Genome Variation Database at 10.6084/m9.figshare.hgv.3651.

## Data Availability

All the data obtained in this study are provided in this report.
